# Basal body structure in *Trichonympha*

**DOI:** 10.1186/s13630-016-0031-7

**Published:** 2016-03-01

**Authors:** Paul Guichard, Pierre Gönczy

**Affiliations:** Department of Cell Biology, University of Geneva, Geneva, Switzerland; Swiss Institute for Experimental Cancer Research (ISREC), School of Life Sciences, Swiss Federal Institute of Technology (EPFL), Lausanne, Switzerland

**Keywords:** *Trichonympha*, Long centriole, Cartwheel, Cryo-tomography

## Abstract

*Trichonympha* is a symbiotic flagellate of many species of termites and of the wood-feeding cockroach. Remarkably, this unicellular organism harbors up to over ten thousand flagella on its surface, which serve to propel it through the viscous environment of the host hindgut. In the 1960s, analysis of resin-embedded *Trichonympha* samples by electron microscopy revealed that the basal bodies that give rise to these flagella are exceptionally long, with a proximal, cartwheel-bearing, region some 50 times longer than that of regular centrioles. In recent years, this salient feature has prompted the analysis of the 3D architecture of *Trichonympha* basal bodies in the native state using cryo-electron tomography. The resulting ~40 Å resolution map of the basal body proximal region revealed a number of novel features that may be conserved in centrioles of other systems. These include proximal–distal polarity of the pinhead structure that links the cartwheel to centriolar microtubules, as well as of the linker between the A and the C microtubules. Moreover, this work demonstrated that the cartwheel is made of stacked ring-like structures that likely each comprise 18 molecules of SAS-6 proteins.

## Organism

*Trichonympha* sp. is an anaerobic symbiotic flagellate, which is part of the parabasalid goup, within the supergroup Excavata [1]. Parabasalids are characterized by parabasal fibers that link their basal bodies to Golgi complexes. *Trichonympha* sp. cells are typically ~100 μm long and lack mitochondria [2]. Note that, unless otherwise stated, the descriptions below pertain to several *Trichonympha* species that share essentially the same basic structural features. The same holds for the related *Pseudotrichonympha* sp., as well as the two Australian species *Deltotrichonympha operculata* and *Koruga bonita* [3–6].

## Basic basal body structure and composition

**Fig. 1:**
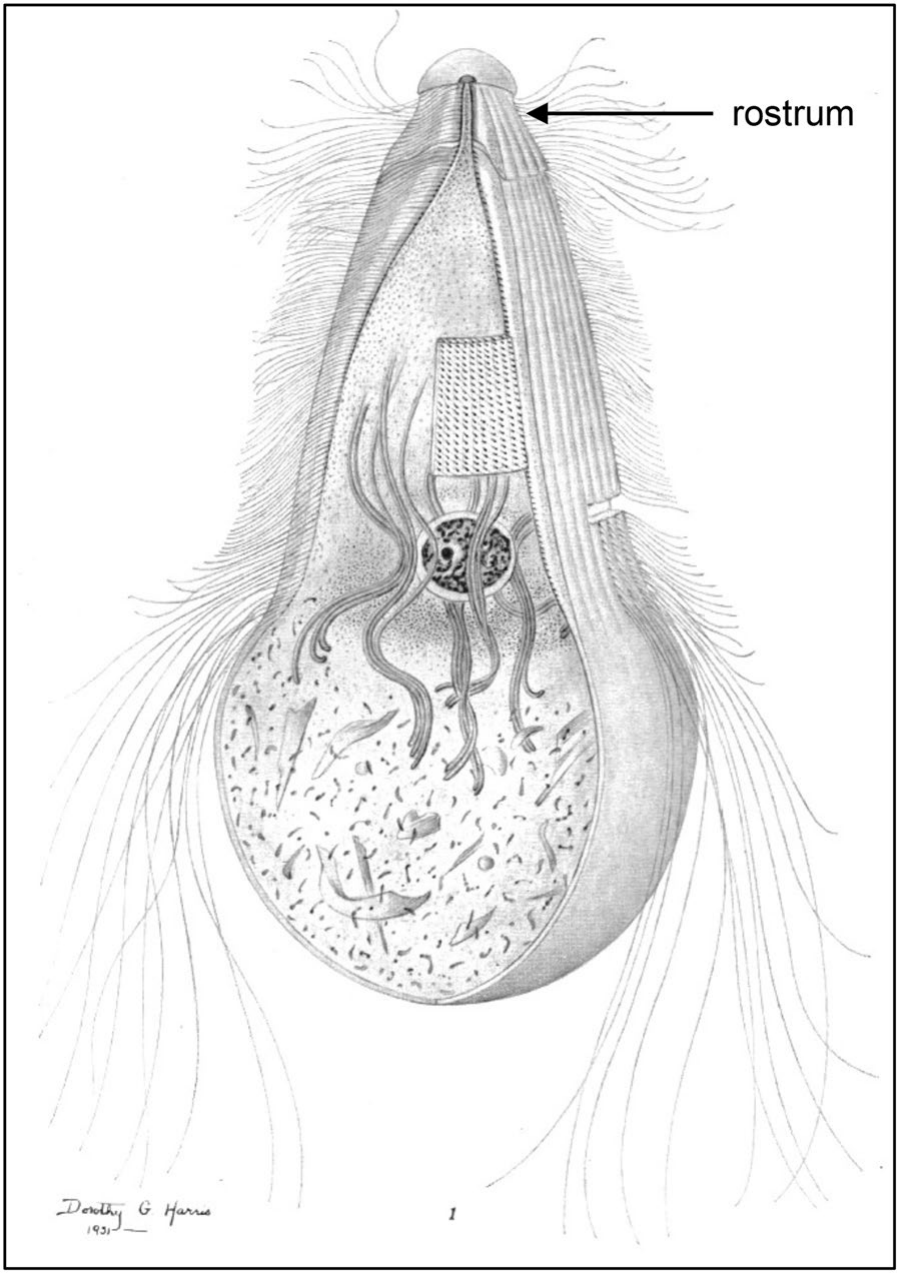
*Trichonympha collaris*. The entire *Trichonympha* cell is covered by flagella, except for the bottom part, where pieces of wood can be seen inside the cell. The rostrum is located at the top of the cell as indicated. A *Trichonympha collaris* cell is approximately 100 μm. Image from Kirby [7]

Canonical microtubule triplets are present in *Trichonympha* basal bodies. Over ten thousand flagella that stem from basal bodies have been estimated to be present on a single *Trichonympha campanula* cell [5, 7] (Fig. 1). In addition, two centrioles are present close to the rostrum (Fig. 1), one long one and one short one [6]. Both delta tubulin and epsilon tubulin are present in the genome of *Trichonympha agilis* [8], although whether the corresponding proteins localize to the basal body is not known.

## Additional basal body structures or accessory structures

**Fig. 2:**
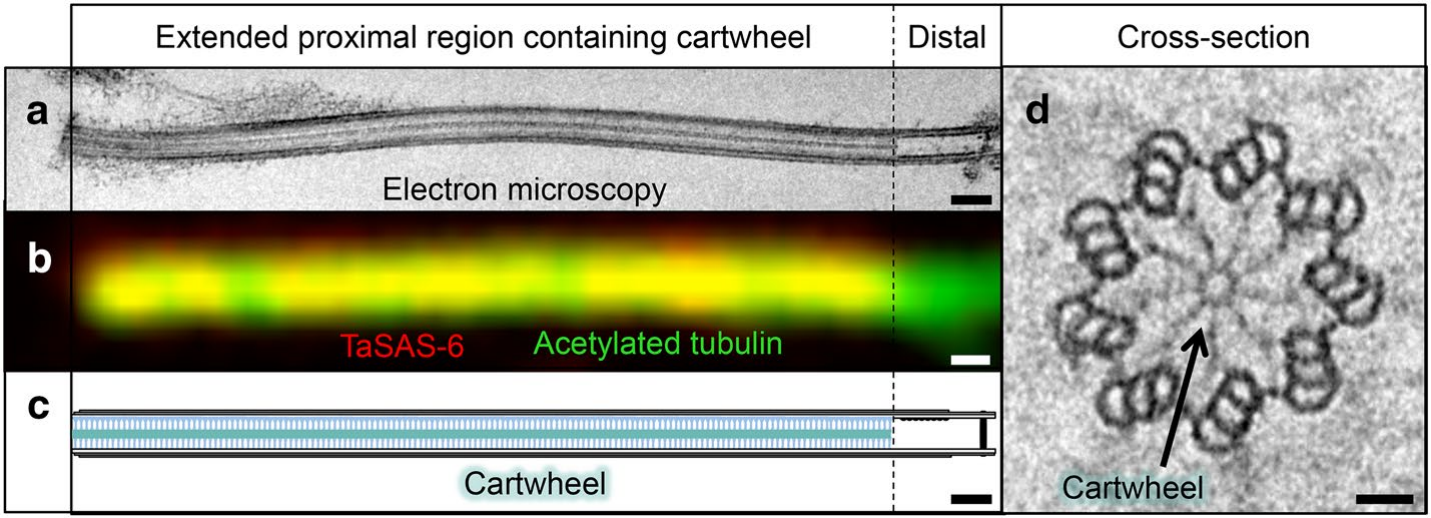
Exceptionally long basal body in *Trichonympha.* **a** Electron micrograph of isolated *Trichonympha* sp. basal body**. b** Immunofluorescence of isolated *Trichonympha* sp. basal body revealing TaSAS-6 localization (*red*, *yellow* in overlay) along the basal body/flagellum complex stained with acetylated tubulin (*green*). **c** Schematic representation of the exceptionally long basal body of *Trichonympha*, with the cartwheel-bearing region highlighted. **d** Cross section of *Trichonympha* basal body; *arrow* points to cartwheel structure, with central hub and nice radial spokes connecting with peripheral microtubules. *Scale bar* in (**a**, **b**) 250 nm, in (**d**) 50 nm

A salient characteristic of *Trichonympha* basal bodies is their unusual length, which results from an extended proximal region (Fig. 2a–c) that bears the cartwheel (Fig. 2d), and which can be up to 5 μm long, instead of the usual 100 nm [5, 8]. Basal body length varies slightly depending on the position in the cell, with basal bodies in the anteriorly located rostrum being shorter than those in the cell posterior [6]. By contrast to the unusually long proximal region, the distal region of *Trichonympha* basal bodies resembles that of other centrioles, including in terms of length, as well as the presence of doublet microtubules and of centrin (Fig. 2a–c) [8, 9]. Distal appendages appear to be present as well, whereas no clear sub-distal appendages have been observed [5]. The first comprehensive ultrastructural analysis of basal bodies in *Trichonympha* using electron microscopy is to be credited to Gibbons and Grimstone [5].

## Basal body origins

How basal bodies form in *Trichonympha* is not known with certainty. In *Deltotrichonympha operculata*, basal bodies are thought to form from short subunits called kinetosomes that are arranged in a columnar fashion in the anterior region of the cell [3, 4]. It is not clear whether basal bodies form de novo or in the vicinity of existing ones.

## Basal body life cycle and other functions

The basal body life cycle has been investigated in *Trichonympha magna* [10], where the long and short centrioles located close to the rostrum seem to act as centrosomes during mitosis, while the basal bodies remain at the cell surface during cell division.

## Identification of basal body components

No proteomic or genomic screen has been performed to date. However, the genome of *Trichonympha agilis* is currently being assembled, which already allowed the identification of mRNAs encoding TaSAS-6 (Fig. 2b), α-tubulin, β-tubulin, δ-tubulin, ε-tubulin [8], as well as centrin (PaG and PiG, unpublished observations).

## Notable basal body findings

**Fig. 3:**
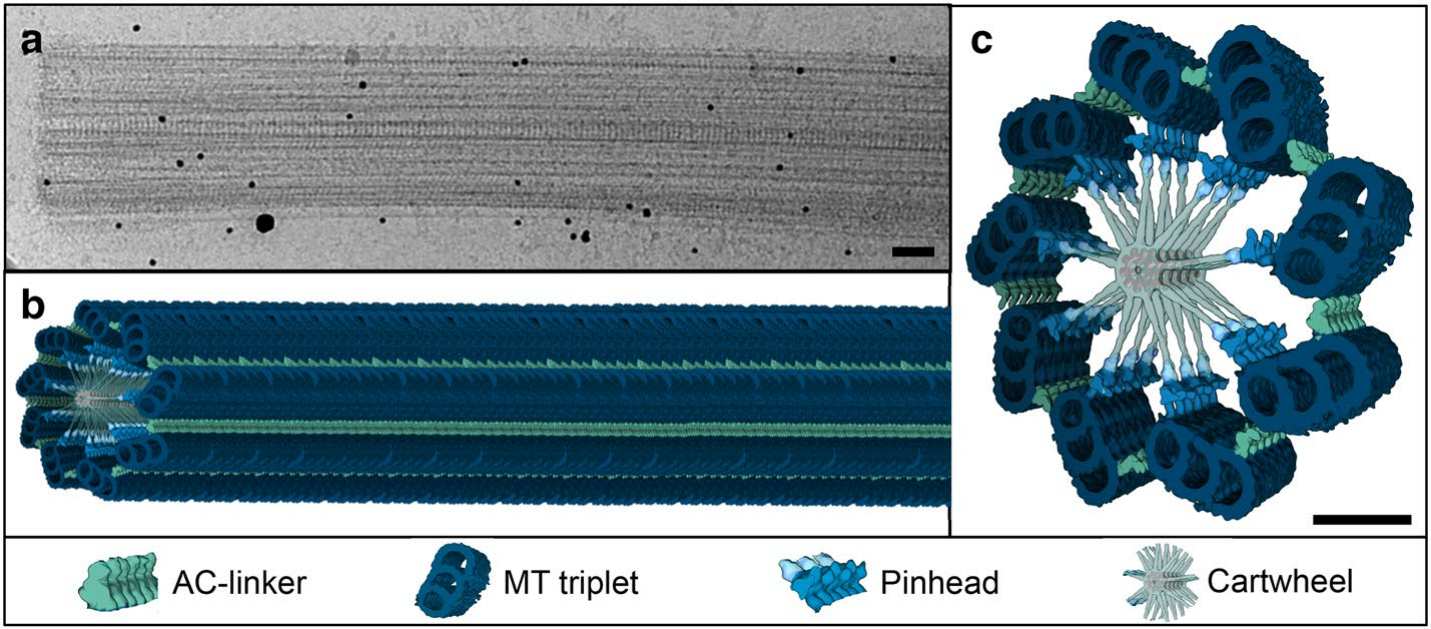
Native structure of the proximal region of *Trichonympha* basal body. **a** Cryo-microscopy image of isolated *Trichonympha* sp. basal body viewed from the side. *Scale bar* 100 nm. **b–c** 3D architecture of the cartwheel-bearing region of basal bodies at ~40 Å resolution, side-view (**b**) and cross section (**c)**. *Scale bar* 50 nm

The 3D architecture of the cartwheel-bearing region of basal bodies in their native state has been elucidated using cryo-microscopy coupled to tomography (Fig. 3a–c) [8]. The resulting ~40 Å resolution map revealed notably the existence of proximal–distal polarity both in the pinhead that links the cartwheel to centriolar microtubules, as well as in the linker situated between the A and the C microtubules (Fig. 3c). Moreover, this analysis demonstrated that the cartwheel comprises a stack of ring-like structures that are ~4.5 nm high and which exhibit a periodicity of ~8.25 nm in the centriole center; by contrast, the periodicity at the periphery is of ~16.5 nm as a result from the merger of spokes from two superimposed rings. This work uncovered also that ring-like assemblies of 18 molecules of SAS-6 proteins that were postulated based on structural work to be at the core of the cartwheel fit well in the cryo-EM map of the *Trichonympha* basal body [8–11].

## Strengths and future of basal body research in *Trichonympha*

One obvious strength of *Trichonympha* as a model system is the large number of basal bodies per cell, which can be easily purified together with the flagella. In addition, the exceptionally long proximal region offers remarkable opportunities for structural analysis by cryo-tomography. However, the anaerobic life cycle and the lack of transgenesis and gene inactivation methods preclude at present functional studies in *Trichonympha*. In the future, *Trichonympha* could be used for proteomic analysis of purified basal bodies, in particular, to uncover proteins specific of the proximal region. Furthermore, the 5-μm-long centriole is a particularly suitable sample for accurate protein localization using super-resolution microscopy.

## Abbreviation

cryo-EM: cryo-electron microscopy
